# From Geometric Exploration to Semantic Completion: Scene Exploration Convolution and Large Format Perception for Adverse-Weather UAV Aerial Object Detection

**DOI:** 10.3390/s26092802

**Published:** 2026-04-30

**Authors:** Yize Zhao, Bo Wang, Jialei Zhan

**Affiliations:** 1 School of Software, Xinjiang University, Urumqi 830008, China; zhaoyize@stu.xju.edu.cn; 2 College of Systems Engineering, National University of Defense Technology, Changsha 410073, China; zhanjialei@nudt.edu.cn

**Keywords:** SELPNet, UAV aerial object detection, adverse weather, scene exploration convolution (SEC), large format perception module (LPM), oriented bounding box (OBB)

## Abstract

Object detection from unmanned aerial vehicle (UAV) imagery is essential for applications such as traffic monitoring, disaster response, and urban surveillance, yet most existing methods are developed and evaluated under clear-sky conditions. In real-world UAV operations, adverse weather including fog, rain, and snow introduces severe image degradation that simultaneously disrupts both the geometric and photometric properties of targets. This paper identifies two fundamental bottlenecks underlying this performance collapse: the lack of geometric invariance in standard convolutional operators and the inability of fixed receptive fields to reconstruct features corrupted by atmospheric interference. To address these bottlenecks, we propose SELPNet (Scene Exploration and Large Format Perception Network), a unified framework that integrates geometric alignment and multi-scale contextual perception into the YOLOv13 head. SELPNet consists of two key modules: (1) The Scene Exploration Convolution (SEC) leverages affine Lie group theory to construct a discrete manifold of rotation and scale transformations, actively probing multiple geometric views and selecting the most coherent response via a Maxout mechanism. (2) The Large Format Perception Module (LPM) introduces a dynamic dilation strategy with depthwise separable convolutions, progressively enlarging the receptive field from fine-grained edge preservation to scene-level contextual perception for semantic completion of degraded regions. We further construct and release AWU-OBB, a large-scale benchmark containing over 18,000 oriented bounding box-annotated UAV images across four representative scene categories. Ablation experiments demonstrate that SEC and LPM yield complementary gains, achieving a combined improvement of +4.26% mAP50 over the YOLOv13-n baseline with only 0.11 M additional parameters and 0.2 extra GFLOPs. The source code will be publicly released upon acceptance of this paper.

## 1. Introduction

Object detection in remote sensing imagery is a fundamental task that supports a wide range of applications, including urban monitoring, disaster response, and traffic management [1,2,3]. Compared with satellite platforms, Unmanned Aerial Vehicles (UAVs) offer lower operational cost, greater deployment flexibility, and high-resolution imaging at low altitudes [4,5,6]. They can rapidly reach target areas and acquire real-time observations from multiple viewpoints. These advantages make UAV-based object detection important for both academic research and practical deployment.

**Figure 1:**
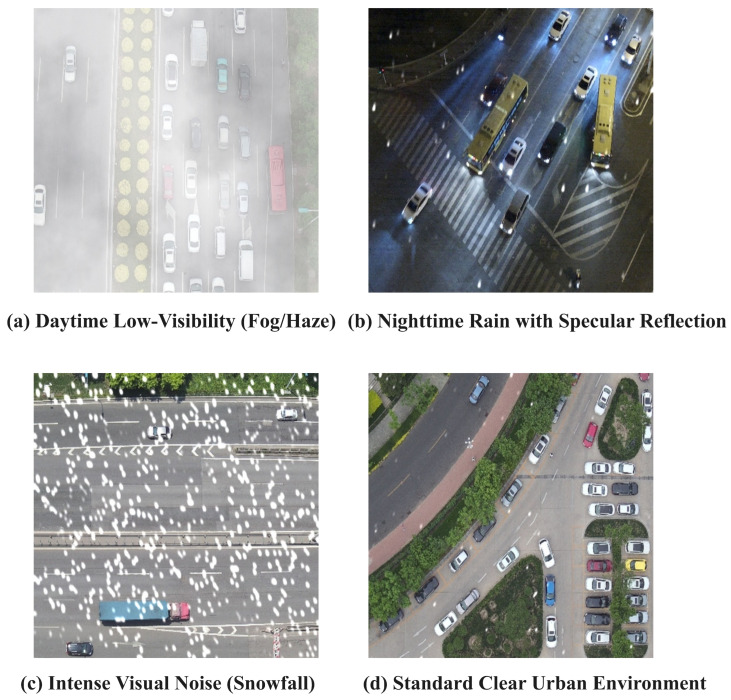
Representative UAV traffic scenes under different environmental conditions: (**a**) daytime low-visibility caused by fog/haze, (**b**) nighttime rain with strong specular reflections, (**c**) snowfall with intense visual noise and partial occlusion, and (**d**) a standard clear urban environment. These examples illustrate how weather and illumination changes degrade contrast, corrupt local details, and increase scene complexity for oriented object detection.

Despite these advantages, most existing studies still focus on clear-sky conditions with stable illumination and high image quality. In real UAV applications, however, adverse conditions such as fog, rain, snow, and nighttime low-light scenes are common rather than exceptional. As illustrated in Figure 1, different environmental conditions introduce distinct forms of image degradation. Fog and haze reduce global contrast through atmospheric scattering and blur the boundary between foreground and background [7,8,9]. In nighttime rainy scenes, wet-road reflections and headlight halos often generate misleading visual responses that interfere with accurate angular estimation. Snowfall and other severe weather may further occlude local target structures and weaken discriminative details [10,11]. Moreover, these degradations are often coupled with flight-induced rotation, scale variation, and motion blur, further increasing the difficulty of reliable detection in UAV imagery [11,12,13]. As a result, conventional detectors are more likely to suffer from localization drift, missed detections, and unstable oriented bounding box (OBB) regression under adverse weather conditions, especially in low-visibility scenes with complex backgrounds.

Existing UAV detectors under adverse weather mainly face two challenges. First, conventional convolution operators lack intrinsic geometric robustness to rotation and scale variation. Under the joint effect of scattering, refraction, and viewpoint changes, target geometry becomes highly unstable, while standard convolutions rely on fixed sampling grids and struggle to learn consistent rotated representations [14,15,16,17]. This limitation is particularly critical for OBB detection, where angular prediction depends heavily on geometric alignment and orientation consistency. Second, fixed receptive fields are insufficient when local image evidence is severely degraded. Once fog, rain, or snow weakens edges, textures, and local contrast, reliable recognition can no longer rely only on local neighborhoods. Without access to broader contextual cues such as road structure, building layout, and scene-level illumination, detectors are more likely to miss targets in low-visibility environments [7,8,18,19].

Although existing studies have improved weather robustness, feature compensation, and multi-scale perception to some extent, most of them address these issues separately and do not provide a unified solution for both geometric instability and contextual incompleteness. In adverse-weather UAV detection, geometric disturbance and semantic corruption often occur simultaneously. Therefore, improving only local feature quality or only contextual aggregation is usually insufficient to prevent performance degradation. This limitation motivates a unified framework that jointly strengthens geometric modeling and contextual perception for robust oriented object detection under complex weather conditions.

To address these challenges, we propose SELPNet, an improved YOLOv13-n framework for UAV oriented object detection under adverse weather. For geometric instability, we design the Scene Exploration Convolution (SEC), which introduces multi-view geometric exploration to improve feature consistency under rotation and scale variation. For degraded local perception and insufficient contextual reasoning, we develop the Large Format Perception Module (LPM), which enlarges the receptive field and strengthens multi-scale contextual modeling to improve semantic compensation in corrupted regions. By integrating SEC and LPM into a unified framework, SELPNet enhances both geometric robustness and contextual perception, thereby improving the reliability of rotated object detection in complex weather conditions.

Beyond model design, the lack of dedicated benchmarks also limits progress in this field. Most existing UAV detection datasets focus on clear-weather scenes and are insufficient for systematic evaluation of oriented object detection under adverse weather. To this end, we construct and open source the AWU-OBB dataset, which contains over 18,000 high-resolution images with oriented bounding box annotations across four representative weather scenarios: fog, rain, snow, and nighttime. More importantly, this dataset is designed not only to validate the proposed method but also to provide a unified benchmark for future research on adverse-weather UAV perception. Experiments on AWU-OBB show that SELPNet improves mAP50 by 4.26% over the YOLOv13-n baseline and achieves superior overall performance on this benchmark.

In summary, the main contributions of this paper are as follows:

- We propose SELPNet, an improved YOLOv13-n framework for UAV oriented object detection under adverse weather, which jointly enhances geometric robustness and contextual perception.
- We design the Scene Exploration Convolution (SEC) to improve feature stability under rotation and scale variation, thereby strengthening the reliability of OBB prediction in degraded UAV imagery.
- We develop the Large Format Perception Module (LPM) to enlarge the receptive field and enhance context-aware semantic compensation in low-visibility scenes.
- We construct the AWU-OBB dataset, a dedicated benchmark with over 18,000 oriented bounding box-annotated UAV images covering four representative adverse-weather scenarios and verify the effectiveness of SELPNet on this benchmark.

The rest of this paper is organized as follows. Section 2 reviews the related work on adverse-weather UAV object detection and feature-driven detection mechanisms. Section 3 presents the overall architecture of SELPNet and details the designs of SEC and LPM. Section 4 describes the AWU-OBB dataset, experimental settings, comparative results, and ablation studies. Section 5 discusses the limitations of the current work and possible future directions. Finally, Section 6 concludes the paper.

## 2. Related Work

### 2.1. UAV Object Detection Under Adverse Weather Conditions

As UAVs are increasingly deployed in adverse environments for surveillance, search and rescue, and environmental monitoring, developing detection algorithms that remain reliable under fog, rain, snow, and low illumination has attracted growing attention [1,6]. Weather-induced degradation, including reduced contrast, color shifts, and random noise [9], blurs object boundaries and causes feature inconsistencies during multi-level aggregation. Existing approaches to this problem broadly fall into three categories: restoration-based preprocessing [12], domain-invariant learning [11], and robust end-to-end detection frameworks [2,4].

Restoration-based methods apply image enhancement before detection. IA-YOLO [12] introduces an image-adaptive module that refines inputs using atmospheric priors, while ELS-YOLO [13] applies configurable enhancement filters for low-illumination scenes. Despite measurable quality improvements, these methods face two practical drawbacks. First, separating visual reconstruction from semantic learning can create conflicts: aggressive denoising or dehazing may remove subtle edge and texture cues required for small-object detection in high-resolution aerial images [9]. Second, the additional inference cost of cascaded restoration modules is prohibitive on UAV edge platforms, where latency constraints are tight.

Domain adaptation methods try to align feature distributions from clean and degraded domains [11]. Munir et al. [11] benchmarked multiple architectures and showed significant performance drops under unforeseen distortions in UAV images. A fundamental weakness of this direction is that it treats weather noise as a uniform domain shift, overlooking the tight coupling between weather-induced occlusion and the geometric variations inherent to aerial perspectives (arbitrary rotation, scale fluctuation). As a result, localization shifts and boundary jitter persist when climatic interference and dynamic flight angles overlap.

More recently, robust end-to-end architectures have attempted to achieve weather resilience without an explicit restoration stage. TPH-YOLOv5 [2] and TPH-YOLOv5++ [4] use Transformer-based prediction heads to strengthen multi-scale context perception. Wang et al. [1] proposed frequency-domain disentanglement to separate environmental noise from structural features, and Kalwar et al. [10] proposed gated differentiable image processing for robust detection in adverse conditions. These models improve robustness against global background noise but still share a common structural weakness: their spatial aggregation and spectral filtering operations lack intrinsic rotation equivariance. When a UAV maneuvers dynamically in fog or rain, the combination of target rotation and weather blur leads to unstable features.

Our framework addresses this gap through the Scene Exploration Convolution (SEC). Rather than relying on data augmentation or computationally heavy attention blocks for orientation handling, SEC embeds affine Lie group constraints directly into the convolution. This provides structural equivariance and feature consistency across rotations and translations at the operator level, producing stable geometric features even in heavily degraded scenes. Combined with a streamlined head, SEC effectively reduces the boundary jitter and angular inconsistency that limit existing UAV detectors in extreme weather.

### 2.2. Feature-Driven Object Detection Mechanisms

Feature extraction quality and multi-scale fusion coherence largely dictate the performance ceiling of modern detectors [8]. Recent studies have further emphasized that high-resolution UAV imagery introduces severe scale variation, dense small-object distribution, and substantial information loss during hierarchical downsampling. To address these issues, multi-scale detection strategies have been extensively explored, including fine-grained feature enhancement, adaptive cross-scale fusion, and density-aware region refinement. Representative examples include MAFDet [20], which improves small-object detection in high-resolution drone images through attention-guided cropped-block refinement, MSUD-YOLO [21], which strengthens multiscale perception for UAV aerial imagery, and AD-Det [22], which explicitly addresses scale variation and class imbalance in UAV-based object detection. These studies further confirm that effective multi-scale representation is a critical prerequisite for robust detection in high-resolution UAV scenes.

In UAV sensing, feature-driven methods have evolved from simple head stacking to architectural designs that optimize receptive fields, align task-specific features, and reconstruct feature pyramids [6,9]. Current work in this area falls into three streams: attention-driven enhancement [3,23,24], task-alignment optimization [14,15], and lightweight architectures [13,25,26,27]. While all three have improved accuracy in general settings, they still face specific problems when applied to aerial imagery: excessive parameter overhead, poor adaptation to arbitrary orientations, and information loss during high-resolution data processing.

Attention-based models use global and local contextual cues to refine feature discrimination. Coordinate Attention [23] encodes cross-channel positional information to recover spatial details lost in standard convolutions. For UAV tasks, SOAM Block [24] introduces scale-and-orientation-aware mechanisms, and Gold-YOLO [3] achieves all-level feature fusion through a Gather-and-Distribute (GD) mechanism. These modules deliver strong perception in cluttered backgrounds, but at the cost of high GFLOPs and parameter counts. On resource-constrained UAV hardware (e.g., NVIDIA Xavier or Jetson Orin), integrating them causes sharp frame rate drops that prevent real-time processing.

Task-alignment methods, represented by TOOD [14] and YOLOX [15], address the conflict between classification and localization branches in one-stage detectors. TOOD dynamically aligns task-specific distributions through a dedicated head and performs well on high-resolution images. However, for aerial targets, these methods tend to prioritize boundary consistency at the expense of fine-grained texture discrimination under background clutter. Furthermore, the information bottleneck during hierarchical transformation, where small-object features are lost during aggressive downsampling, remains problematic. YOLOv9 [25] partially mitigates this with Programmable Gradient Information (PGI), but the trade-off between feature preservation and efficiency is not fully resolved.

Lightweight architectures such as YOLOv10 [27] and ELS-YOLO [13] redesign information flow for efficient perception. YOLOv10 uses a dual-label assignment strategy for NMS-free detection, and ELS-YOLO employs a lightweight feature selection pyramid for low-light aerial scenes. These models achieve fast inference but tend to sacrifice deep semantic integration, leading to poor recall for densely distributed small targets. A perception module that handles large-format aerial data without heavy computational burden is still needed.

Our framework introduces the Large Format Perception Module (LPM) to fill this gap. In contrast to the parameter-heavy modules in Gold-YOLO [3], LPM uses a structured bottleneck with depthwise separable convolutions, specifically tailored to UAV computational constraints. This design preserves high-level semantic richness during feature pyramid processing while keeping the parameter footprint small, allowing critical small-target features to pass through feature transformation without significant loss. Together with SEC, LPM enables the improved YOLOv13 to achieve a favorable balance between perception accuracy and deployment efficiency for large-scale aerial scenarios.

The discussion above highlights two complementary but so far unaddressed challenges. Weather-resilient methods improve environmental robustness yet fail to account for arbitrary target rotations, leading to geometric instability in dynamic aerial views. Feature-driven methods offer advanced multi-scale perception but remain too computationally expensive for real-time UAV deployment. Our improved YOLOv13 framework SELPNet integrates SEC and LPM to address both problems at once: SEC provides geometric invariance at the operator level, while LPM supplies efficient large-format contextual perception. The following section details the specific design of each module.

Relevant work summaries are presented in Table 1.

## 3. Methods

**Figure 2:**
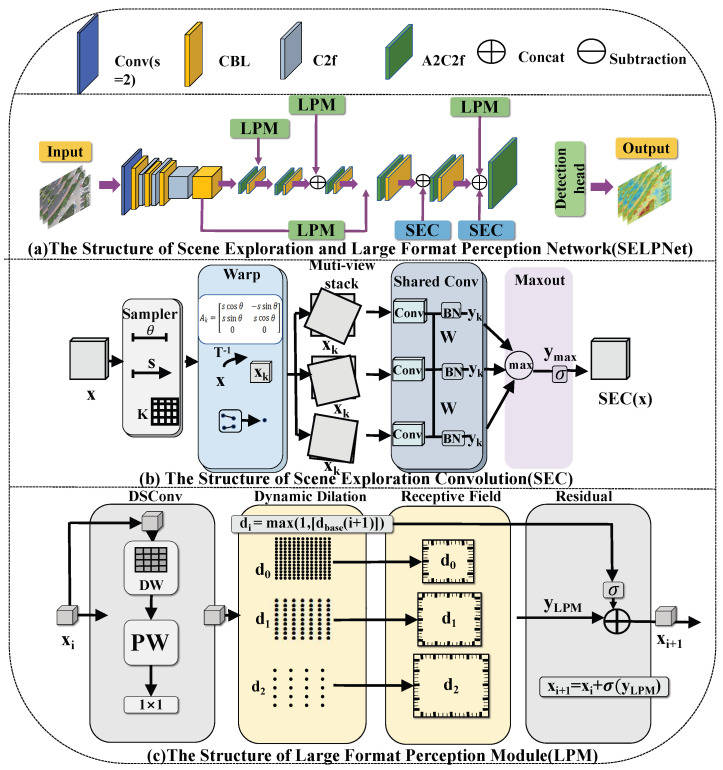
Architecture of the SELPNet and its core components. (**a**) The overall structure of SELPNet. (**b**) The structure of Scene Exploration Convolution (SEC). (**c**) The structure of Large-format Perception Module (LPM).

SELPNet is a unified detection framework built upon YOLOv13-n, in which the Large-format Perception Module (LPM) and the Scene Exploration Convolution (SEC) are jointly integrated into the detection head. The framework first extracts multi-scale features from the input image using the baseline backbone and neck. On top of this foundation, LPM is inserted into four feature fusion and refinement bottlenecks in the head to enhance contextual perception and multi-scale representation under degraded visual conditions. SEC is introduced into two transition branches of the detection head, where it replaces standard convolutions to perform geometry-aware feature exploration before final multi-scale prediction. In this design, LPM mainly improves contextual perception and semantic compensation, whereas SEC strengthens geometric robustness and feature consistency under rotation, occlusion, and background interference. Their collaborative integration within the same detection pipeline enables SELPNet to provide more reliable support for oriented object detection in adverse-weather UAV imagery. Figure 2 illustrates the overall architecture and the two core components of SELPNet.

### 3.1. Baseline: YOLOv13-n

YOLOv13-n is adopted as the baseline architecture in this work. It represents a compact detection framework tailored for edge devices and embedded deployment on Unmanned Aerial Vehicle (UAV) platforms. As a recent variant in the YOLO family, YOLOv13-n follows the standard end-to-end detection pipeline, which includes a backbone for feature extraction, a neck for multi-scale fusion, and a head for final inference. At the same time, the network structure is carefully simplified to satisfy the real-time processing demands of high resolution remote sensing imagery.

Regarding model scale and computational complexity, YOLOv13-n applies a compound scaling strategy. In the Nano configuration, the depth coefficient is set to 0.50, the width coefficient is set to 0.25, and the maximum number of channels is limited to 1024. This configuration preserves essential non-linear representation capacity while reducing redundant computation. Such a balance is particularly important for UAV platforms with constrained hardware resources, as it lowers parameter size and shortens inference time during onboard deployment.

In the detection head, four DSC3k2 blocks in the baseline configuration, located at lines 31, 36, 42, and 47, are replaced with DSC3k2a modules to instantiate the LPM strategy. As the implementation form of LPM, DSC3k2a introduces dynamic dilation into the original bottleneck structure, enabling stronger contextual perception and multi-scale feature refinement under degraded visual conditions.

In addition, two standard convolution layers in the head transition branches, located at lines 40 and 45, are replaced with ALCGonv modules to implement the SEC strategy. By introducing geometry-aware feature exploration before the final multi-scale prediction stages, SEC strengthens feature consistency and geometric robustness under rotation, occlusion, and background interference.

For feature aggregation, YOLOv13-n integrates the HyperACE module, known as Advanced Context Enhancement, together with the FullPAD_Tunnel mechanism for feature routing. HyperACE fuses shallow spatial details with deeper semantic information across multiple scales, including P4, P6, and P8, so that both small-object localization and large-object classification can be handled effectively. The FullPAD_Tunnel module maintains consistent information transmission during multi-path fusion through dedicated padding and alignment operations. Finally, a unified Detection head performs object classification and oriented bounding box regression on the P3, P4, and P5 feature maps.

Based on these characteristics, YOLOv13-n is adopted as the structural foundation of our method due to its efficient inference speed and stable multi-scale detection capability. However, under adverse weather conditions, limited viewpoints, and complex scene distributions, the original architecture still suffers from insufficient contextual compensation and unstable geometric representation. To address these limitations, SELPNet introduces LPM and SEC as two targeted head-level enhancements to the baseline network, thereby improving robustness in challenging meteorological environments.

### 3.2. Scene Exploration Convolution (SEC)

Extracting robust features is particularly challenging for object detection on UAV platforms. Unlike terrestrial surveillance, UAV sensors face complex geometric variations: arbitrary rotations, scale fluctuations, and perspective distortions caused by rapid maneuvers and altitude changes. In adverse weather (dense fog, heavy rain, or low light), atmospheric scattering and motion blur further obscure target boundaries, compounding the geometric difficulty. Standard CNNs provide translation invariance through their grid-based sampling but cannot explicitly model rotation or scale equivariance; instead, they must learn these transformations implicitly with redundant parameters. To address this limitation, we propose the Scene Exploration Convolution (SEC), a geometry-aware operator grounded in affine Lie group theory, which actively probes and aligns multi-perspective features within the YOLOv13 head.

To approximate continuous geometric transformations within a discrete search space, the SEC module begins by sampling rotation angles $\theta_{i}$ and scale factors $s_{j}$. This discretization defines the search manifold G tailored for UAV-borne imagery:

(1) $$\theta_{i}=-\theta_{\max}+\frac{2i}{N_{r}-1}\theta_{\max},\;i\in \left\{0,\ldots ,N_{r}-1\right\}$$

(2) $$s_{j}=s_{\min}+\frac{j}{N_{s}-1}\left(s_{\max}-s_{\min}\right),\;j\in \left\{0,\ldots ,N_{s}-1\right\}$$

where $N_{r}$ and $N_{s}$ represent the sampling frequencies for rotation and scaling, respectively.

Each parameter pair $\left(\theta_{i},s_{j}\right)$ is mapped to a unique transformation index $k=i\cdot N_{s}+j$, where k∈{1,…,K} and $K=N_{r}\times N_{s}$. The corresponding 2×3 affine transformation matrix $A_{k}$ is formulated as:

(3) $$A_{k}=\left[\begin{array}{ccc} s_{j}\cos\theta_{i} & -s_{j}\sin\theta_{i} & 0\\ s_{j}\sin\theta_{i} & s_{j}\cos\theta_{i} & 0 \end{array}\right]$$

To align target features with convolutional kernels, we employ inverse warping to map each target coordinate (u,v) back to the source coordinate $\left(u_{k}^{\prime },v_{k}^{\prime }\right)$ on the input feature map *x*:

(4) $$\left(\begin{array}{c} u_{k}^{\prime }\\ v_{k}^{\prime } \end{array}\right)=A_{k}\left(\begin{array}{c} u\\ v\\ 1 \end{array}\right)$$

The warped feature map $x_{k}$ is then generated via bilinear interpolation to ensure differentiability:

(5) $$x_{k}\left(u,v\right)=\mathrm{Bilinear}\left(x,u_{k}^{\prime },v_{k}^{\prime }\right)$$

A shared learnable kernel W is applied across all warped feature maps to maintain parameter efficiency. The transformation-invariant response is distilled by selecting the maximum activation across the entire affine set G:

(6) $$y_{k}=\mathrm{BN}\left(W*x_{k}\right)$$

(7) $$y_{\max}=\max\left\{y_{1},y_{2},\ldots ,y_{K}\right\}$$

The final output of the SEC module is obtained by applying a non-linear activation σ:

(8) $$\mathrm{SEC}\left(x\right)=\sigma \left(y_{\max}\right)$$

The SEC module produces geometrically stabilized features that are more resistant to distortion-induced noise. In SELPNet, SEC is implemented by replacing two standard convolution layers in the transition branches of the detection head with ALCGonv modules, thereby enhancing geometric consistency before final multi-scale prediction. This design is particularly beneficial under adverse weather conditions, where rotation, occlusion, and background interference often destabilize local feature representations. In the overall framework, SEC does not function as a standalone precursor to LPM in a strictly sequential manner; instead, it operates collaboratively with LPM within the same detection pipeline. SEC primarily strengthens geometric robustness, whereas LPM focuses on contextual perception and semantic compensation.

In practice, the sampling density should remain moderate: insufficient sampling weakens geometric coverage, whereas excessively dense sampling increases branch redundancy and interpolation-induced ambiguity, which may reduce the effectiveness of Maxout-based response selection.

### 3.3. Large Format Perception Module (LPM)

In UAV-based object detection, images captured under dense fog, heavy precipitation, or low-light transitions often suffer from severe contrast attenuation, edge diffusion, and background noise. Under such conditions, conventional convolution operators with fixed receptive fields (RFs) mainly focus on local textural cues and are unable to adequately capture the broader structural context required for robust recognition. To address this limitation, we propose the Large Format Perception Module (LPM) to improve feature reconstruction under adverse visual degradation. In the detection head, LPM is instantiated by replacing DSC3k2 blocks with DSC3k2a modules, which introduce a dynamic dilation perception mechanism for context-aware feature refinement.

At its core, LPM employs Depthwise Separable Convolution (DSConv) to enlarge receptive fields while maintaining low computational cost. The FLOPs of DSConv can be expressed as:

(9) $${\mathrm{FLOPs}}_{\mathrm{DSConv}}=H\times W\times C_{\mathrm{in}}\times \left(k^{2}+C_{\mathrm{out}}\right)$$

To facilitate hierarchical multi-scale feature extraction, we define $x_{i}^{\prime }$ as the intermediate representation produced by the first DSConv layer within the *i*-th bottleneck. Meanwhile, a dynamic dilation rate $d_{i}$ is introduced to progressively enlarge the receptive field with network depth:

(10) $$x_{i}^{\prime }=\sigma \left(\mathrm{BN}\left(\mathrm{DSConv}\left(x_{i}\right)\right)\right)$$

(11) $$d_{i}=\max\left(1,\left\lfloor d_{\mathrm{base}}\times \left(i+1\right)\right\rfloor \right),\;i\in \left\{0,\ldots ,n-1\right\}$$

where $d_{\mathrm{base}}$ denotes the fundamental dilation base.

With the dynamically adjusted dilation rate, LPM performs context-aware feature refinement over the intermediate representation:

(12) $$y_{\mathrm{LPM}}=\mathrm{BN}\left(W{*}_{d_{i}}x_{i}^{\prime }\right)$$

where ${*}_{d_{i}}$ denotes convolution with dilation rate $d_{i}$. The refined feature is then injected into the current bottleneck output through a residual update:

(13) $$x_{i+1}=x_{i}+\sigma \left(y_{\mathrm{LPM}}\right)$$

This residual formulation preserves spatial stability during hierarchical feature reuse while progressively expanding contextual perception. When haze, rain, or snow partially obscures object boundaries, the enlarged receptive field enables semantic completion by exploiting broader environmental cues, such as road contours and surrounding spatial layout, to compensate for missing local evidence. As the implementation form of LPM, DSC3k2a inherits the efficient gradient flow of the original bottleneck design while introducing large-range contextual perception and stronger degradation awareness. As a result, the enhanced detection head achieves more reliable feature refinement and perceptual consistency across diverse adverse-weather UAV scenarios.

## 4. Experiments

### 4.1. Datasets

To evaluate the robustness of the proposed framework for UAV oriented object detection under adverse weather, we constructed a large-scale dedicated dataset named AWU-OBB (Adverse Weather UAV Oriented Bounding Box). Unlike mainstream remote sensing benchmarks such as DOTA, HRSC2016, and DIOR-R, which mainly capture scenes under favorable illumination and clear visibility, AWU-OBB is specifically designed for UAV perception in adverse weather and low-visibility environments. Its primary objective is to support oriented vehicle detection under challenging meteorological conditions from UAV viewpoints, which also gives the dataset clear practical relevance in special real-world application scenarios.

Prior work has already introduced the IMC dataset [28], and subsequent studies [29] have further advanced weather-robust UAV perception in this line of research.Feature-reconstruction-based few-shot detection research in remote sensing further suggests that discriminative feature reconstruction is important when annotated samples or reliable visual evidence are limited [30]. These efforts confirm the practical importance of adverse-weather UAV sensing. However, the goal of constructing AWU-OBB is not to replace existing benchmarks but to provide a more task-aligned benchmark for the specific problem studied in this paper, namely UAV oriented object detection under adverse weather conditions. In particular, compared with IMC and other related datasets, AWU-OBB places greater emphasis on four representative weather-induced degradation patterns, including fog/haze, nighttime rain with specular reflection, snowfall-induced visual noise, and standard clear urban scenes used as a control subset. More importantly, AWU-OBB adopts oriented bounding box (OBB) annotations, which are better suited for characterizing target orientation variation, elongated aspect ratios, and dense object layouts under UAV viewpoints. In addition, AWU-OBB focuses on more diverse urban and peri-urban traffic scenes, such as multi-lane roads, signalized intersections, roadside parking areas, and open paved areas near built-up structures. Therefore, AWU-OBB is designed not only to validate the proposed method but also to provide a more targeted benchmark for future research on adverse-weather UAV oriented object detection.

Built for evaluating UAV object detection in complex all-weather environments, AWU-OBB mainly covers representative traffic scenes in urban and peri-urban areas, including multi-lane road segments, signalized intersections, roadside parking areas, and open paved regions near built-up structures. The imagery was acquired from low-altitude UAV viewpoints, with flight heights ranging from 25 m to 35 m. Detailed scene-wise statistics of AWU-OBB are summarized in Table 2, including the number of images, annotated vehicle instances, and the train/validation/test split for each representative weather scenario.

Overall, AWU-OBB contains 18,195 high-resolution aerial images, including 12,680 images for training, 1947 for validation, and 3568 for testing. The dataset includes five finely annotated vehicle categories commonly found in urban-road and highway scenes: car, truck, bus, van, and freight car. Considering the high object density, large aspect-ratio variations, and arbitrary target orientations in UAV imagery, all instances are annotated with Oriented Bounding Boxes (OBBs) in the format of (x,y,w,h,θ). Compared with horizontal bounding boxes, this annotation strategy provides a more accurate representation of target geometry and effectively reduces background interference in low-visibility conditions. All annotations were completed through manual labeling to ensure annotation quality and geometric consistency.

In total, AWU-OBB contains 236,392 annotated vehicle instances across the five categories. Among them, car accounts for 207,427 instances, truck for 10,451, bus for 6144, van for 5914, and freight car for 6456.

**Figure 3:**
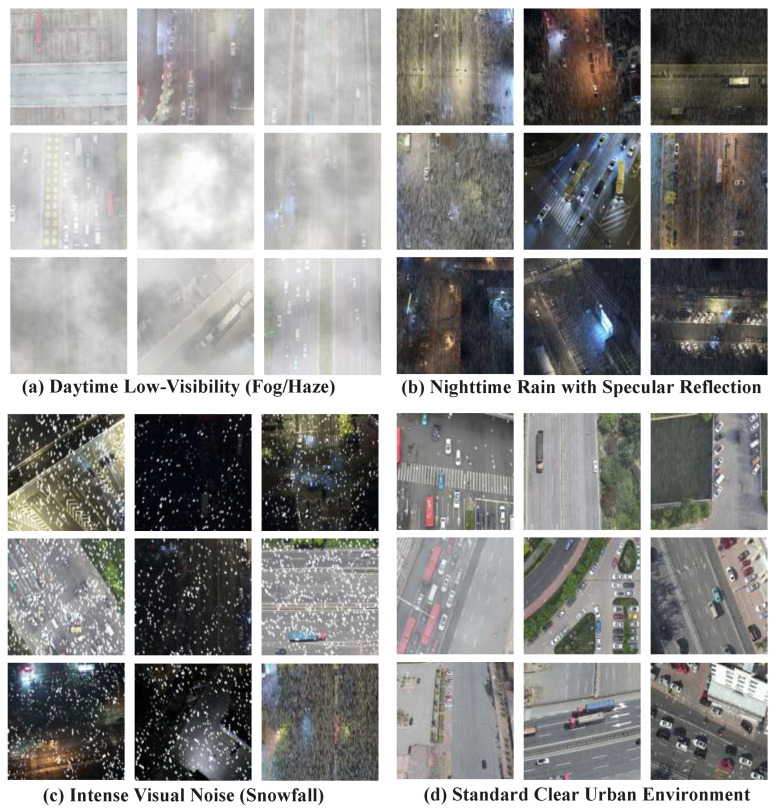
Representative samples from the proposed AWU-OBB dataset, categorized into four representative scene categories: (**a**) Daytime Low-Visibility (Fog/Haze) with severe contrast attenuation and atmospheric scattering; (**b**) Nighttime Rain with Specular Reflection, characterized by wet-road reflections and headlight halos; (**c**) Intense Visual Noise (Snowfall), where dense high-frequency noise causes target occlusion; and (**d**) Standard Clear Urban Environment, which serves as the control subset.

A key strength of AWU-OBB lies in its diverse range of weather-induced degradations and scene complexities. Based on visual degradation characteristics and practical interference patterns, the dataset is divided into four representative scene categories, as illustrated in Figure 3:

- **Daytime Low-Visibility (Fog/Haze):** Dominated by dense fog and haze, these images exhibit severe contrast attenuation and strong atmospheric scattering, which blur object boundaries and suppress local visual details. This category evaluates the model’s ability to preserve global semantic perception when local evidence is heavily degraded.
- **Nighttime Rain with Specular Reflection:** This category combines low illumination with wet-road reflections caused by nighttime rain. Specular glare and headlight halos from vehicles often generate strong false responses, making it a critical test of detection consistency under complex lighting and reflective backgrounds.
- **Intense Visual Noise (Snowfall):** Characterized by snowfall-induced high-frequency random noise, this category introduces severe occlusion and structural corruption over target regions. It is designed to assess the model’s capability to recover and identify meaningful geometric cues under strong visual interference.
- **Standard Clear Urban Environment:** This category consists of standard clear-weather urban scenes and serves as a control subset. It is used to verify whether the proposed method maintains strong detection performance under normal conditions while improving robustness in adverse-weather scenarios.

The AWU-OBB dataset is strictly partitioned into training, validation, and test sets to support fair evaluation. During preprocessing, all images preserve their original aspect ratios and are resized to a unified input resolution of 640×640 pixels. This setting follows the standard input protocol of the YOLOv13 framework and ensures a fair comparison across different methods under a unified computational budget. At the same time, we acknowledge that resizing high-resolution UAV images to 640×640 may discard some fine-grained local details, especially under adverse-weather conditions where discriminative cues are already weakened by fog, rain, or snow. This setting provides a favorable balance between detection accuracy and inference efficiency.

In addition, the unified preprocessing pipeline establishes a consistent data foundation for both the main experiments and the ablation studies. Overall, AWU-OBB more realistically reflects the practical challenges of UAV oriented object detection under adverse weather and offers an application-driven benchmark for evaluating related methods.

### 4.2. Experimental Settings

To ensure the reproducibility of the experimental results, all training and evaluation procedures were conducted on the AutoDL cloud computing platform. The hardware configuration included an NVIDIA GeForce RTX 4090D GPU with 24 GB VRAM, an AMD EPYC 9754 128-Core Processor with 18 allocated vCPUs, and 60 GB of system memory. The software environment consisted of Ubuntu 22.04, Python 3.12, and PyTorch 2.5.1, with hardware acceleration provided by CUDA 12.4. This setup ensured efficient processing of the AWU-OBB dataset, which contains 12,680 training images.

**Figure 4:**
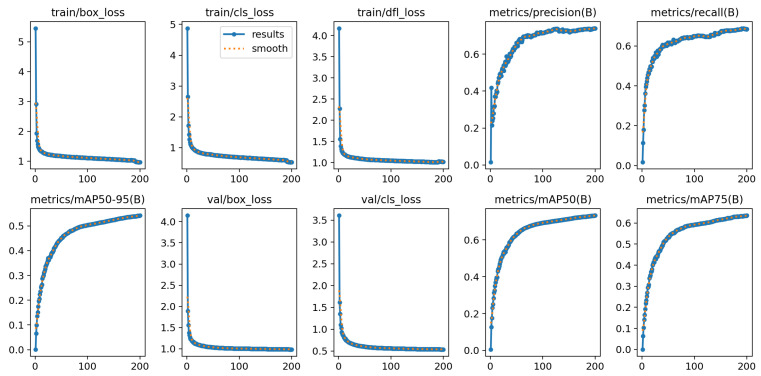
Training curves of SELPNet on the AWU-OBB dataset over 200 epochs. The training and validation losses decrease steadily, while precision, recall, and mAP metrics improve consistently, indicating stable convergence of the proposed model.

The model was trained in an end-to-end manner for 200 epochs with a batch size of 16. The input image resolution was fixed at **640×640** pixels. We employed the Stochastic Gradient Descent (SGD) optimizer with an initial learning rate ($lr_{0}$) of 0.01, a momentum of 0.937, and a weight decay of 0.0005. To stabilize early-stage optimization, a warmup strategy was applied during the first 3 epochs. As shown in Figure 4, SELPNet exhibits a stable optimization process on AWU-OBB over the 200-epoch training schedule. The training and validation losses decrease steadily, while precision, recall, and mAP metrics improve consistently.

The training objective consisted of bounding box regression loss, classification loss, and distribution focal loss (DFL). For oriented bounding box (OBB) inference, the confidence threshold was set to 0.25, and the IoU threshold for Rotated Non-Maximum Suppression (R-NMS) was set to 0.7. Following the standard YOLO training protocol, Mosaic augmentation was disabled during the final 10 epochs to improve boundary refinement. Detailed hardware and hyperparameter settings are summarized in Table 3.

### 4.3. Main Results

Table 4 summarizes the quantitative comparison results of different object detection methods on the AWU-OBB dataset. All compared methods were evaluated under the same data split and training protocol, while their backbone configurations followed the official or widely adopted implementations. Overall, SELPNet achieves the best detection performance on the AWU-OBB benchmark and shows a clear improvement over the YOLOv13-n baseline.

Beyond the compared detection baselines in Table 4, recent remote-sensing studies have also investigated texture-aware representation learning, heterogeneous binary pixel-difference modeling, and low-data-dependence network design [40,41,42], further supporting the importance of robust feature construction for challenging remote-sensing interpretation tasks.

Among the one-stage detectors, RetinaNet [31] achieves 52.14% mAP50 with a ResNet-50 backbone and 54.37% with ResNet-101, indicating that a deeper backbone brings moderate gains in degraded aerial imagery but at the cost of significantly more parameters (55.2 M vs. 36.3 M). Similarly, TOOD [14] improves from 56.73% (ResNet-50) to 58.91% (ResNet-101) and FCOS [32] from 53.82% to 55.48%. These methods mainly rely on fixed-scale anchor designs or anchor-free mechanisms with standard convolutional backbones, and their performance drops substantially under the severe degradation present in AWU-OBB even with deeper feature extractors. IA-YOLO [12] improves mAP50 to 60.22% by introducing image-adaptive preprocessing. TPH-YOLOv5 [2], Gold-YOLO [3], and TPH-YOLOv5++ [4] further improve detection performance, reaching 62.91%, 63.47%, and 64.05% mAP50, respectively, although these gains are accompanied by relatively high parameter counts and computational cost.

Among the two-stage and query-based detectors, the effect of backbone depth is also clearly observed. Faster R-CNN [33] achieves 48.23% mAP50 with ResNet-50 and 50.36% with ResNet-101, while Cascade R-CNN [34] improves from 55.71% to 57.84% under the same backbone upgrade. DINO [35] attains 62.13% mAP50 with ResNet-50 and further reaches 63.87% with ResNet-101, indicating stronger contextual modeling capability than conventional two-stage detectors. However, these methods generally require substantially higher parameter budgets (up to 88.6 M for Cascade R-CNN with ResNet-101) and FLOPs (up to 157.3 G for DINO with ResNet-101), which limits their practicality for lightweight UAV deployment. The consistent but moderate gains from deeper backbones across all these methods suggest that backbone depth alone is insufficient to address the fundamental challenges of adverse-weather UAV detection.

The YOLO-based detectors form the most representative comparison group in this study because they provide a favorable balance between detection accuracy and model efficiency. From YOLOv6 [26] to YOLOv10-n [27], the performance on AWU-OBB improves steadily, with mAP50 increasing from 59.43% to 65.84%. YOLOv11-n [39] further extends this trend to 66.53% mAP50, while the latest YOLOv26-n [38] achieves 70.18% mAP50 with 2.81 M parameters and 7.1 GFLOPs, representing the strongest baseline among existing YOLO variants. The YOLOv13-n baseline achieves 69.04% mAP50 with only 2.45 M parameters and 6.4 GFLOPs, providing a compact yet competitive foundation for subsequent improvement.

Built upon this baseline, SELPNet further improves mAP50 to 73.30%, exceeding YOLOv13-n by 4.26 percentage points and surpassing the latest YOLOv26-n by 3.12 percentage points, achieving the best result among all compared methods. Under the stricter mAP metric averaged over IoU thresholds from 0.50 to 0.95, SELPNet reaches 54.22%, which is 4.08 percentage points higher than the baseline and 2.85 percentage points above YOLOv26-n. At the mAP75 threshold, SELPNet attains 63.62%, improving upon the baseline value of 59.32% by 4.30 percentage points. These results indicate that the proposed SEC and LPM modules effectively enhance geometric representation stability and contextual compensation under complex meteorological conditions, and that such task-specific enhancements outperform the general architectural advances introduced in newer YOLO generations.

**Figure 5:**
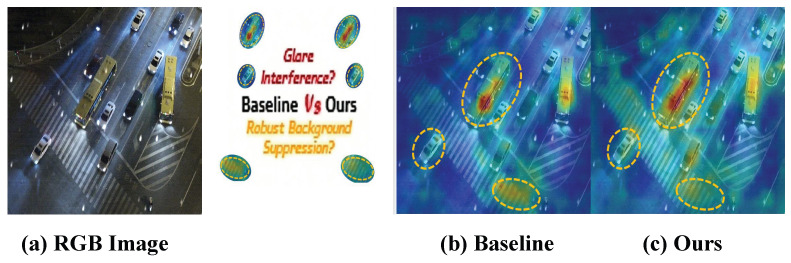
Qualitative comparison of feature attribution between the YOLOv13-n baseline and the proposed SELPNet under an adverse-weather UAV scene. (**a**) Input RGB image. (**b**) Feature-attribution map of the YOLOv13-n baseline. (**c**) Feature-attribution map of the proposed SELPNet. In the attribution maps, warmer colors (e.g., red and yellow) indicate stronger feature responses, whereas cooler colors (e.g., blue and green) indicate weaker responses. Compared with the baseline, SELPNet produces more concentrated responses around target vehicles and exhibits stronger suppression of spurious activations in reflective and cluttered background regions, demonstrating more discriminative target-focused representation under adverse-weather conditions.

To further illustrate the effectiveness of the proposed method, Figure 5 presents a qualitative comparison of feature attribution between the YOLOv13-n baseline and SELPNet under a challenging adverse-weather UAV scene. Compared with the baseline, SELPNet produces more concentrated responses in target regions and suppresses background interference more effectively, especially in the presence of rain streaks, wet-road reflections, and complex nighttime illumination. This observation is consistent with the quantitative improvements reported in Table 4.

In terms of model size, SELPNet contains only 2.56 M parameters and 6.6 GFLOPs, introducing an increase of merely 0.11 M parameters and 0.2 GFLOPs over YOLOv13-n. Notably, this is even more compact than YOLOv26-n (2.81 M parameters, 7.1 GFLOPs) while achieving substantially higher accuracy. This compact overhead suggests that the proposed modules improve detection accuracy while maintaining a lightweight model profile. Such parameter efficiency benefits from the shared-kernel design in SEC and the computational efficiency of depthwise separable convolution in LPM.

These results indicate that SELPNet maintains strong overall robustness on the AWU-OBB benchmark under diverse adverse-weather conditions. Compared with methods that rely on degradation-specific adaptation or additional preprocessing stages, SELPNet improves detection performance through a unified design that combines geometric stabilization and contextual perception enhancement. Specifically, SEC strengthens feature consistency under rotation and scale variation, while LPM expands the effective receptive field to exploit broader scene cues when local evidence is degraded. This complementary design helps the model achieve reliable overall detection performance in complex UAV adverse-weather scenarios without resorting to heavy backbone configurations.

### 4.4. Ablation Study

Validation of the Effectiveness of Geometric Modelling and Strategy Optimisation. Table 5 and the accompanying visual results demonstrate the individual and combined contributions of our proposed modules to the YOLOv13-Nano baseline.

First, the integration of the Scene Exploration Convolution (SEC) yields a significant improvement of +2.75% mAP50, raising the baseline performance to 71.79%. This gain stems from the affine Lie group convolution mechanism embedded within SEC. By actively generating multi-view geometric transformations via affine grids and aggregating the most robust features through a Maxout strategy, the network acquires explicit geometric invariance. This capability effectively mitigates boundary jitter caused by arbitrary rotations and scale fluctuations inherent in UAV aerial imagery.

Second, the Large-field Perception Module (LPM) delivers the most substantial individual gain of +3.70% mAP50, reaching 72.74%. This validates the efficacy of the Dynamic Dilation strategy implemented within the DSC3k2a block. Unlike fixed dilation rates, our approach linearly increases the dilation factor across stacked bottleneck layers to construct a hierarchical multi-scale receptive field. This design captures long-range dependencies, which is particularly beneficial for detecting weak-textured targets in complex environments, while maintaining a low computational footprint of approximately 6.4 GFLOPs.

Finally, the combination of SEC and LPM achieves a peak performance of 73.3% mAP50, representing a total gain of +4.26% mAP50. This result highlights the complementary nature of the two strategies, where SEC rectifies local geometric distortions and LPM expands the semantic context. Crucially, this synergistic effect is achieved with negligible increases in parameters (+0.11 M) and computational cost (+0.2 GFLOPs), demonstrating a high efficiency-to-performance ratio suitable for resource-constrained UAV platforms.

**Figure 6:**
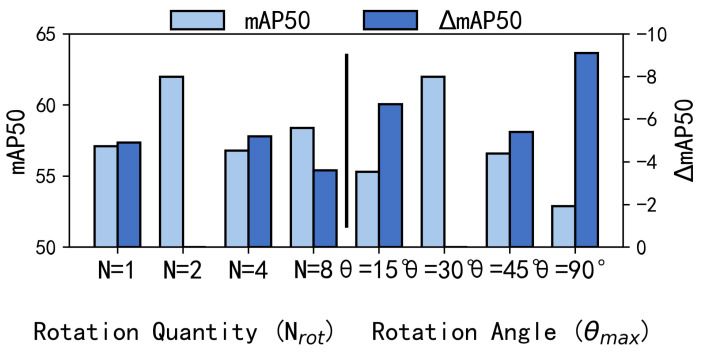
Comparison of variants regarding number of rotations ($N_{rot}$) and maximum rotation angle ($\theta_{max}$).

**Figure 7:**
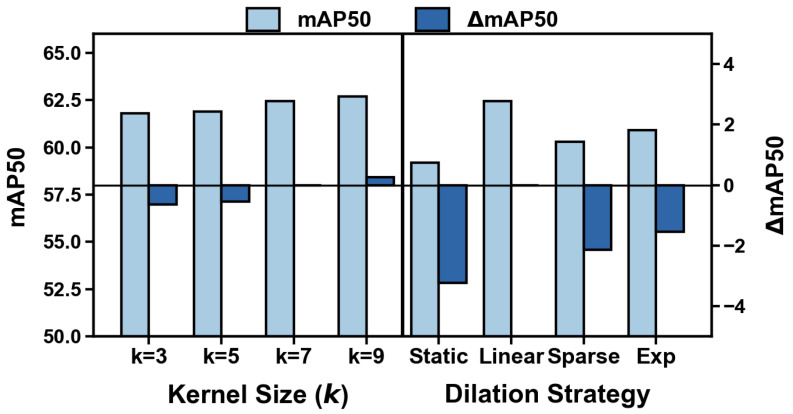
Comparison of variants regarding Kernel Size (*k*) and Dilation Strategy.

Unless otherwise specified, the main experiments follow the 200-epoch training schedule described in Section 4.2. For computational efficiency, however, the internal ablation studies in Table 6 and Table 7 and Figure 6 and Figure 7 were trained for 50 epochs only, as their purpose is to evaluate the relative contributions of different design choices under a consistent setting rather than to obtain fully converged final performance. Therefore, the ablation results in this section should be interpreted as evidence of relative design trends under a controlled training budget, rather than as definitive conclusions about the fully converged optimum of each configuration.

Table 6 and Figure 6 show that there exists a “moderate optimality” for both the number of rotations ($N_{\mathrm{rot}}$) and the maximum rotation angle ($\theta_{\max}$) in terms of detection performance.

For the number of rotations, the results suggest a balance between geometric coverage and redundancy: $N_{\mathrm{rot}}=2$ delivers the best accuracy (62.00% mAP50). Too few rotations ($N_{\mathrm{rot}}=1$) provide insufficient geometric diversity (−4.90%), whereas too many rotations ($N_{\mathrm{rot}}=4/8$) yield limited benefits and even degrade performance (−5.20%/−3.60%), indicating that excessive rotation views may introduce feature ambiguity or noise rather than informative orientation cues.

For the maximum rotation angle, a similar “moderate optimality” is observed. $\theta_{\max}=30{}^{\circ}$ achieves the highest mAP50, consistent with typical orientation jitter in remote sensing objects. A smaller angle range (15°) is insufficient to cover larger pose variations, while overly large angles (45° and 90°) bring in substantial background interference and invalid priors, leading to a pronounced performance drop (up to −9.10%).

This trend can be explained by the discrete-search nature of SEC. When the sampling frequency is too low, the affine search manifold is too sparse to adequately cover the geometric variations caused by UAV viewpoint changes, target rotation, and scale fluctuation, so the module may fail to align targets with a sufficiently coherent transformed view. However, when the sampling frequency becomes excessively high, neighboring affine branches become strongly redundant, and repeated bilinear interpolation over densely sampled transformations may introduce smoothing effects and response ambiguity. Under such conditions, the Maxout selection is more likely to face competition among highly similar branches, which weakens the discriminative advantage of multi-view exploration. Therefore, a moderate sampling frequency provides the best trade-off between geometric coverage and branch redundancy.

Impact of Receptive Field and Dynamic Dilation: Table 7 and Figure 7 illustrate the ablation results for the internal design of the DSC3k2a module. For the kernel size (*k*), Group A reveals a trade-off between context capture and computational efficiency. Small kernels (k=3) yield suboptimal accuracy (61.80%) due to insufficient contextual information, whereas increasing *k* to 7 significantly boosts the mAP50 to 62.44%. Although the extra-large kernel (k=9) achieves a slightly higher performance (62.70%), the marginal gain (+0.26%) does not justify the increased computational cost, indicating that k=7 represents the optimal balance. Regarding the dilation strategy (Group B), the proposed Linear growth strategy achieves the best performance (62.44%), surpassing the Static baseline (59.20%) by a substantial margin (+3.24%). Notably, overly aggressive expansion strategies, such as Sparse and Exponential, lead to performance degradation (60.30% and 60.90%, respectively). This suggests that while expanding the receptive field is critical, maintaining spatial continuity is equally important to avoid the loss of fine-grained local features essential for detecting small remote sensing targets.

### 4.5. Scene-Wise Performance Analysis Under Diverse Adverse Conditions

Table 8 presents the scene-wise performance comparison on the AWU-OBB test set. SELPNet consistently surpasses YOLOv13-n under all four scene categories, including clear, fog-haze, nighttime rain, and snowfall, which demonstrates the superior robustness of the proposed method under diverse urban adverse-weather conditions. In particular, SELPNet achieves the best results in the clear scene while still maintaining stable gains in challenging degraded environments. The improvements under fog-haze and nighttime rain indicate that SELPNet is more resistant to low visibility, illumination variation, and specular interference. Notably, even in the snowfall scene, which suffers from severe visual noise and partial target corruption, SELPNet still yields clear advantages over the baseline. Moreover, the consistent gains in both mAP50 and mAP75 suggest that the proposed method improves not only detection recall but also precise oriented localization quality. These results verify that SELPNet generalizes well across heterogeneous observation conditions and is better suited for practical UAV-based vehicle detection in complex real-world scenarios.

### 4.6. Lightweight Variant Analysis

To further investigate the trade-off between detection accuracy and computational efficiency, we design a lightweight variant of the proposed framework, termed SELPNet-Lite. This variant explores the minimal effective configuration of the SEC and LPM modules, aiming to achieve improved detection performance over the baseline while reducing both parameter count and computational cost.

The design of SELPNet-Lite involves two targeted simplifications. For the SEC module, the lightweight variant SEC-Lite reduces the discrete affine search space by collapsing the rotation and scale sampling to a single learnable affine view (K=1). Instead of exhaustively enumerating $N_{r}\times N_{s}$ transformation branches and selecting the maximum response via Maxout, SEC-Lite parameterizes the rotation angle $\hat{\theta }$ and scale factor $\hat{s}$ as trainable scalar parameters that are optimized jointly with the network weights through standard backpropagation. This reformulation replaces the combinatorial search with gradient-based geometric adaptation, eliminating the multi-branch forward passes that constitute the primary computational bottleneck of the full SEC module. Formally, the learnable affine matrix is defined as:

(14) $$A_{\mathrm{lite}}=\left[\begin{array}{ccc} \hat{s}\cos\hat{\theta } & -\hat{s}\sin\hat{\theta } & 0\\ \hat{s}\sin\hat{\theta } & \hat{s}\cos\hat{\theta } & 0 \end{array}\right]$$

where $\hat{\theta }$ and $\hat{s}$ are initialized to 0 and 1.0, respectively. The SEC-Lite output is then:

(15) $$\mathrm{SEC}\text{-}\mathrm{Lite}\left(x\right)=\sigma \left(\mathrm{BN}\left(W*\mathrm{Bilinear}(x,A_{\mathrm{lite}})\right)\right)$$

For the LPM module, the lightweight variant LPM-Lite reduces the number of DSC3k2a bottleneck layers from four to two and decreases the depthwise separable convolution kernel size from k=7 to k=5. This configuration preserves the core dynamic dilation mechanism while substantially reducing the number of dilated convolution operations. The dilation schedule follows the same linear growth rule $d_{i}=\max\left(1,\left\lfloor d_{\mathrm{base}}\times \left(i+1\right)\right\rfloor \right)$ but operates over fewer stages, resulting in a more compact receptive field hierarchy.

Table 9 presents the comparison between the baseline, SELPNet-Lite, and the full SELPNet.

SELPNet-Lite achieves 71.51% mAP50, improving upon the baseline by 2.47 percentage points. At the same time, it reduces the parameter count to 2.38 M (−0.07 M compared to the baseline) and the computational cost to 6.2 GFLOPs (−0.2 GFLOPs) while increasing the inference speed to 58.73 FPS (+3.31 FPS). This improvement in both accuracy and efficiency is attributed to two factors. First, SEC-Lite replaces multi-branch exhaustive search with a single learnable affine transformation, which eliminates redundant forward passes and reduces latency while retaining the capacity to adapt to the dominant geometric distortion in the data through gradient optimization. Second, LPM-Lite with fewer bottleneck stages and smaller kernels introduces less computational overhead than the original DSC3k2a configuration, yet the preserved dynamic dilation mechanism still provides meaningful receptive field expansion beyond the baseline.

Compared with the full SELPNet (73.30% mAP50), SELPNet-Lite trades 1.79 percentage points in accuracy for a substantially lower latency (58.73 vs. 32.69 FPS) and a smaller model footprint. This trade-off is particularly relevant for deployment on resource-constrained UAV edge platforms such as NVIDIA Jetson series, where real-time inference under strict power budgets is a practical requirement. The results suggest that the proposed SEC and LPM modules support flexible scaling: the full configuration delivers the best accuracy for offline or server-side analysis, while the lightweight variant provides a deploy-friendly alternative that still surpasses the baseline across all evaluation metrics.

### 4.7. Model Deployment and Edge-Device Validation

To assess the balance between detection accuracy and inference latency in practical deployment scenarios, we compared SELPNet against all representative detection frameworks evaluated in this study. As shown in Table 10, experiments were conducted using the AWU-OBB dataset with input images resized to 640×640. To further contextualize the computational cost, we additionally include two large backbone-based configurations that represent the upper bound of model capacity.

Our proposed SELPNet, built on the compact CSPDarknet backbone with only 2.56 M parameters, achieves the highest mAP50 of 73.30% and mAP of 54.22%, outperforming all other compared models. Its per-image inference time of 30.59 ms on the RTX 4090D platform serves as the baseline (1.00×) for evaluating runtime efficiency.

Among the one-stage detectors, RetinaNet and FCOS with ResNet-50/101 backbones run slightly faster (21.14–26.18 ms, 0.69–0.86×) but deliver substantially lower accuracy, with mAP50 ranging from 52.14% to 55.48%. TOOD (ResNet-101) achieves comparable latency (30.67 ms, 1.00×) yet falls behind SELPNet by 14.39% mAP50. Weather-adapted models such as IA-YOLO (19.12 ms, 0.63×) and ELS-YOLO (20.37 ms, 0.67×) benefit from faster inference but remain 13.08% and 12.43% below SELPNet in mAP50. Heavier one-stage models including TPH-YOLOv5 (28.01 ms), Gold-YOLO (26.04 ms), and TPH-YOLOv5++ (29.59 ms) achieve latencies comparable to SELPNet but lag behind by 10.39%, 9.83%, and 9.25% in mAP50 respectively, indicating that their heavy attention and multi-head designs do not translate into proportional accuracy gains for adverse-weather UAV detection.

Two-stage and query-based detectors consistently exhibit longer inference times. Faster R-CNN with ResNet-50/101 requires 33.56–39.37 ms (1.10–1.29×) while achieving only 48.23–50.36% mAP50. Cascade R-CNN (ResNet-50/101) and DINO (ResNet-50/101) reach 37.74–45.25 ms (1.23–1.48×)—up to 1.48× slower than SELPNet—yet their highest mAP50 of 63.87% (DINO, ResNet-101) is still 9.43% lower than that of SELPNet. This highlights the computational inefficiency of such heavy-backbone detection paradigms in practical UAV deployment.

Among the YOLO-based lightweight detectors, YOLOv6 through YOLOv13-n achieve faster inference speeds (14.64–18.05 ms, 0.48–0.59×) owing to their sub-3M parameter scales. However, even the strongest among them, YOLOv26-n (70.18% mAP50), still falls behind SELPNet by 3.12 percentage points. The lightweight variant SELPNet-Lite achieves 71.51% mAP50 at 17.03 ms (0.56×), surpassing YOLOv26-n in both accuracy and speed with fewer parameters (2.38 M vs. 2.81 M). These results confirm that the proposed SEC and LPM modules introduce meaningful accuracy gains without disproportionate latency overhead.

More notably, large backbone-based detectors such as Cascade R-CNN with Swin-L and DINO with Swin-L experience a dramatic increase in latency, reaching 152.95 ms and 122.36 ms, respectively—4 to 5 times slower than SELPNet—despite offering comparable mAP50 values of 72.08% and 72.35%, which are still lower than that of SELPNet (73.30%). This reveals the fundamental limitation of scaling up backbone capacity for adverse-weather UAV detection: the marginal accuracy gains from large-scale vision transformers do not justify the prohibitive increase in computational cost, especially on resource-constrained UAV platforms where inference efficiency is a critical deployment requirement.

Edge-device deployment on Raspberry Pi 4. To further validate the deployability of our approach on edge devices, we implemented SELPNet on a Raspberry Pi 4 Model B (Raspberry Pi Ltd., Cambridge, UK) platform (Broadcom BCM2711, quad-core Cortex-A72 @ 1.8 GHz, 8 GB LPDDR4 RAM). The model was exported from PyTorch to ONNX format and executed using the ONNX Runtime inference engine with CPU-only computation. Input images were resized to 640×640 following the same preprocessing protocol as the main experiments. Table 11 summarizes the deployment results.

On the Raspberry Pi 4 platform, the YOLOv13-n baseline achieves a per-image inference time of 1.38 s, which serves as the 1.00× reference for edge-device efficiency comparison. One-stage detectors such as TOOD (ResNet-101) and TPH-YOLOv5++ require 12.05 s and 18.91 s respectively—8.7 to 13.7 times slower—while achieving substantially lower mAP50 (58.91% and 64.05%). Two-stage detectors are even more prohibitive: Cascade R-CNN (ResNet-101) reaches 22.73 s (16.47×) and DINO (ResNet-101) takes 19.48 s (14.12×), yet their best mAP50 (63.87%) remains 9.43% below that of SELPNet. The large parameter footprints (51.0–92.3 M) of these architectures further exacerbate memory pressure on the 8 GB ARM platform.

Notably, SELPNet-Lite achieves 1.31 s per image (0.95×)—faster than the YOLOv13-n baseline (1.38 s)—while improving mAP50 by 2.47 percentage points. This advantage stems from the single-view learnable affine design of SEC-Lite, which eliminates multi-branch overhead, combined with the reduced DSC3k2a stages in LPM-Lite. The full SELPNet achieves 1.52 s per image (1.10×), with a latency increase of approximately 10% relative to YOLOv13-n, while delivering the highest mAP50 of 73.30% among all compared methods.

More notably, large backbone-based detectors such as Cascade R-CNN (Swin-L, 253.7 M parameters) and DINO (Swin-L, 218.4 M parameters) require 68.42 s and 54.19 s per image respectively—40 to 50 times slower than the YOLOv13-n baseline—yet their mAP50 values of 72.08% and 72.35% are still lower than that of SELPNet (73.30%). These results demonstrate that scaling up backbone capacity is not a viable strategy for edge deployment in adverse-weather UAV scenarios.

Despite limited computational resources, SELPNet maintains competitive detection accuracy with practical inference latency suitable for low-frequency or offline edge analysis. The modular design of SEC and LPM supports flexible accuracy–speed trade-offs: the full SELPNet delivers the best accuracy for offline or batch-processing UAV edge applications, while SELPNet-Lite provides a deploy-friendly alternative that surpasses the latest YOLOv26-n in both accuracy and speed. These results confirm the deployability of the proposed framework on resource-constrained platforms for UAV-based environmental monitoring and post-mission analysis scenarios, where per-frame latency requirements are less stringent than those of continuous real-time video streams.

### 4.8. Visualization

**Figure 8:**
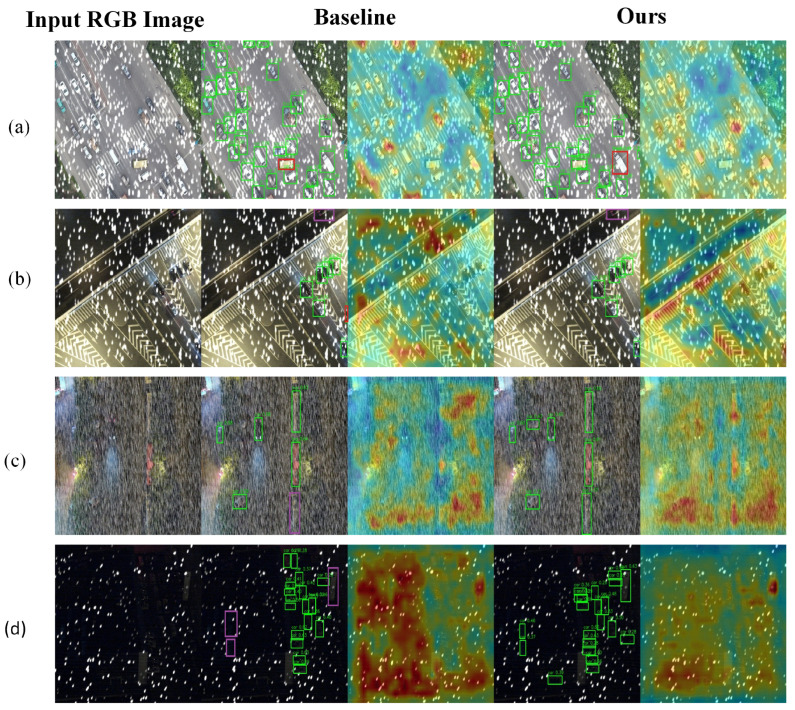
Qualitative comparison in snowy conditions. (**a**) A snowy scene with dense local visual noise over the road area. (**b**) A snowy scene with strong high-frequency interference and partially obscured vehicles. (**c**) A snowy scene with severe snowfall-induced texture corruption. (**d**) A low-illumination snowy scene with dense target distribution. For each row, the columns show the input RGB image, the YOLOv13-n baseline result, and the proposed SELPNet result. Green bounding boxes indicate correctly detected targets (true positives, TP), red boxes indicate false positives (FP), and purple boxes indicate missed detections (false negatives, FN). High-density snowfall introduces high-frequency random noise that disrupts local object textures and structural contours. This visualization demonstrates the localization accuracy and robustness of the proposed method under snowy conditions.

**Figure 9:**
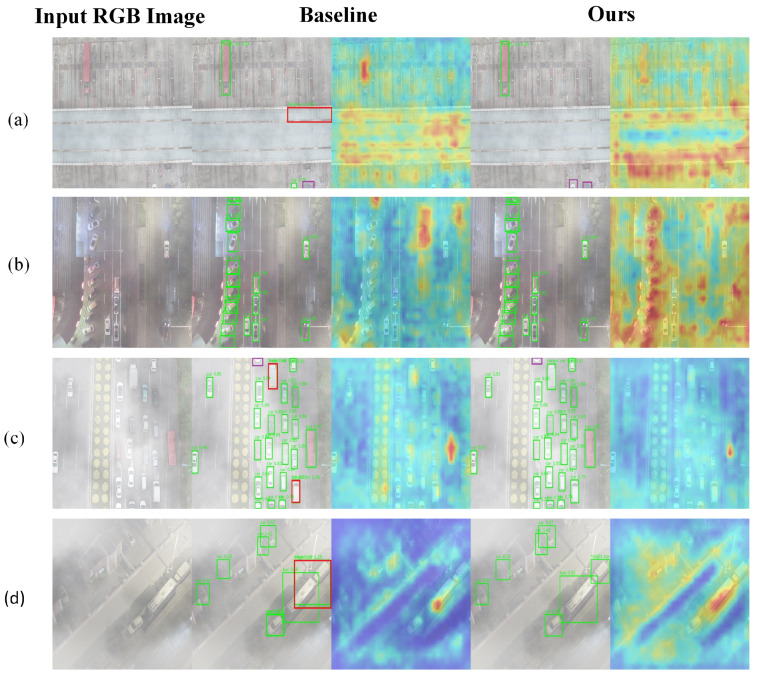
Qualitative comparison in fog-haze conditions. (**a**) A low-visibility foggy scene with weak target contrast. (**b**) A foggy scene with dense vehicles and strong atmospheric scattering. (**c**) A foggy urban traffic scene with blurred object boundaries. (**d**) A severely degraded fog-haze scene with partially obscured vehicles. For each row, the columns show the input RGB image, the YOLOv13-n baseline result, and the proposed SELPNet result. Green bounding boxes indicate correctly detected targets (true positives, TP), red boxes indicate false positives (FP), and purple boxes indicate missed detections (false negatives, FN). Fog and haze reduce global contrast and blur object boundaries, making vehicle localization more difficult. This visualization shows that the proposed method produces more compact and accurate bounding boxes under low-visibility conditions.

**Figure 10:**
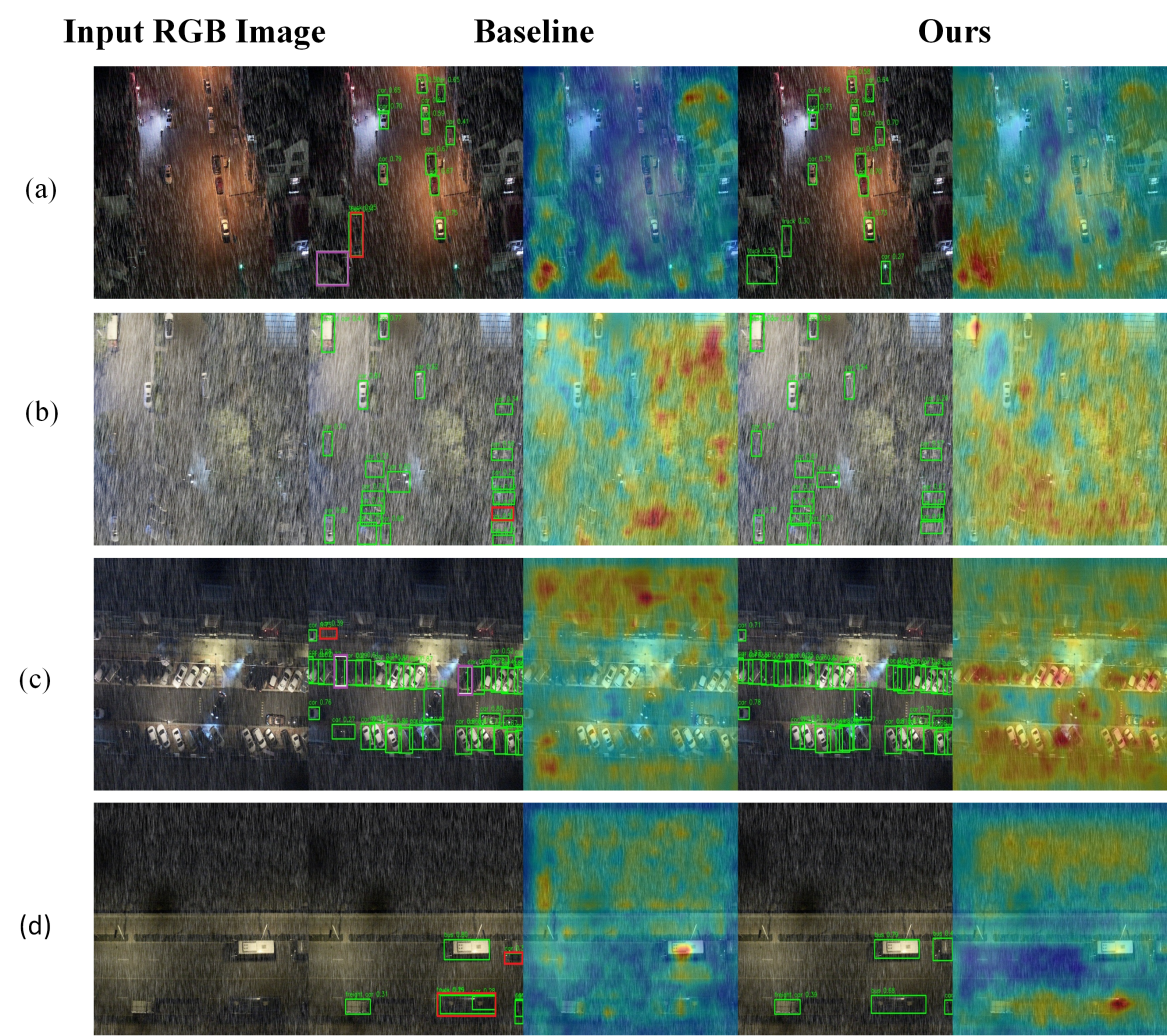
Qualitative comparison in nighttime rainy conditions with specular reflection. (**a**) A nighttime rainy scene with wet-road reflection and vehicle light halos. (**b**) A low-illumination scene with strong rain-induced visual interference. (**c**) A nighttime road scene with dense vehicle lights and reflective background regions. (**d**) A dark roadside scene with sparse vehicles and severe illumination imbalance. For each row, the columns show the input RGB image, the YOLOv13-n baseline result, and the proposed SELPNet result. Green bounding boxes indicate correctly detected targets (true positives, TP), red boxes indicate false positives (FP), and purple boxes indicate missed detections (false negatives, FN). Complex specular reflections and vehicle light halos introduce optical artifacts that interfere with accurate detection. This visualization demonstrates the proposed method’s capability to suppress optical noise and maintain precise oriented bounding boxes under nighttime rainy conditions.

**Figure 11:**
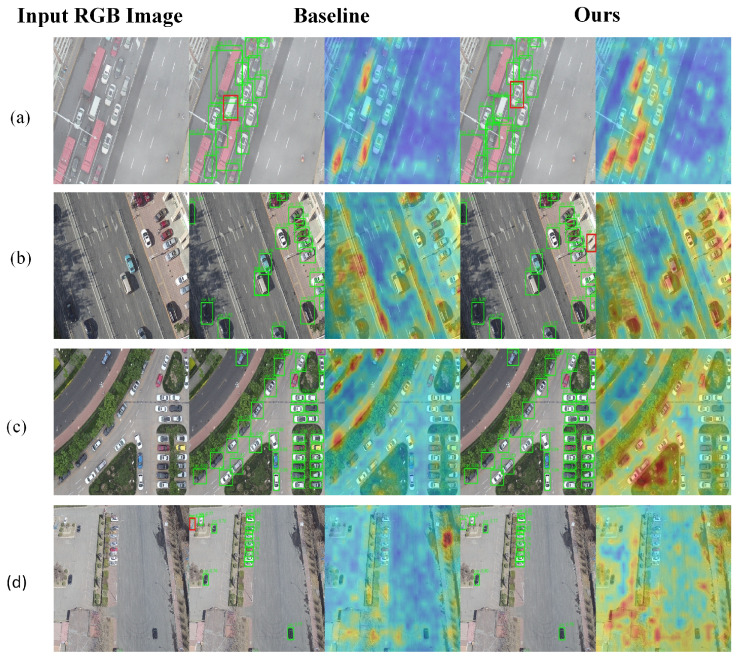
Qualitative comparison in clear urban environments. (**a**) A clear urban road scene with dense traffic targets. (**b**) A clear parking-area scene with closely adjacent vehicles. (**c**) A standard urban road-intersection scene with dense vehicle distribution. (**d**) A clear open-road scene with sparse and medium-scale vehicles. For each row, the columns show the input RGB image, the YOLOv13-n baseline result, and the proposed SELPNet result. Green bounding boxes indicate correctly detected targets (true positives, TP), and red boxes indicate false positives (FP). Although physical degradation is minimal, dense object distributions still challenge feature extraction and localization. This visualization shows improved bounding-box compactness and accurate localization of vehicles in standard urban scenes.

To deeply evaluate the perceptual stability and robustness of the proposed YOLOv13 framework under diverse environmental conditions, we conducted a qualitative analysis across four representative scenarios: snowy, foggy, nighttime, and clear urban environments. By comparing the attention heatmap activations and oriented bounding box (OBB) accuracy between the baseline and our proposed method, we validate the technical advantages of our modules in mitigating complex meteorological interference, as illustrated in Figure 8, Figure 9, Figure 10 and Figure 11.

In the snowy scenario shown in Figure 8, high-density snowfall introduces high-frequency random noise that directly disrupts local object textures and structural contours. The baseline model exhibits significant perceptual bias, producing diffuse and irregular heatmap activations in boundary regions, which results in noticeable angle drift and boundary jitter. In contrast, the Scene Exploration Convolution (SEC) module performs structured manifold search via Lie group theory to suppress geometric noise, while the Large Format Perception Module (LPM) expands the receptive field to reconstruct obscured contours. Visualizations confirm that our method achieves tighter and more precise bounding box fitting, significantly reducing the probability of localization failure caused by high-frequency noise.

In the foggy scenario shown in Figure 9, atmospheric scattering significantly degrades global contrast and blurs feature edges, making it difficult for the baseline to extract deep semantic information. The baseline heatmap displays large and irregular activation patterns, leading to frequent missed detections of distant or low-contrast targets. By optimizing feature pyramid propagation, our LPM module establishes robust global contextual associations based on road topology and environmental surroundings, even under extreme gradient loss. Our method anchors the heatmap focus precisely on vehicle keypoints and maintains accurate OBB boundaries, demonstrating superior semantic completion and contextual reasoning capabilities.

In the nighttime scenario shown in Figure 10, complex specular reflections on wet surfaces and vehicle light halos create severe optical artifacts that interfere with detection. The baseline model is highly susceptible to these artifacts, often resulting in significant geometric distortion of the detection responses. Leveraging the affine invariance derived from Lie group theory, our model accurately filters out these irrelevant responses from dynamic perspectives. The heatmaps show that while the baseline produces false activations outside vehicle contours, our method suppresses light halo noise effectively, ensuring that detected bounding boxes align precisely with the principal axes of the vehicles.

In the standard clear environment shown in Figure 11, although physical degradation is minimal, the baseline model often struggles with insufficient feature identification in dense parking areas, resulting in loose bounding box fitting or overlapping edges. By enhancing the fusion of deep semantic features with shallow spatial details, our model generates sharper and more compact bounding boxes that better encapsulate vehicles of varying sizes. This improvement is particularly evident in dense regions, where our model maintains high angular perception accuracy and effectively eliminates the boundary jitter observed in the baseline approach, achieving high-fidelity geometric fitting.

Although the proposed method consistently outperforms the baseline in all four scenarios, several failure cases can still be observed. Under extremely dense snowfall or severe low-visibility conditions, a few distant or heavily occluded vehicles remain difficult to localize reliably, especially when local structural cues are strongly corrupted. In nighttime scenes, strong specular reflections and headlight halos may still trigger local false responses in highly reflective road regions. In clear urban scenes, dense parking layouts and closely adjacent vehicles may occasionally cause missed detections or imperfect OBB alignment. These cases suggest that, while SELPNet improves both geometric robustness and contextual perception, highly complex degradation, dense object interactions, and severe local occlusion still pose challenges for reliable UAV-oriented detection.

In summary, the visual comparisons across these four scenarios confirm that the geometric robustness provided by the SEC module and the multi-scale contextual awareness provided by the LPM module establish a solid foundation for robust all-weather UAV object detection.

## 5. Limitations and Future Steps

Several limitations of the current method should be acknowledged. First, SEC adopts a fixed rotation and scale sampling strategy. Although this discrete design is convenient for implementation, it may still limit modeling flexibility in scenes with more complex geometric variations. The lightweight variant SELPNet-Lite partially addresses this issue by replacing the exhaustive search with a learnable affine parameterization, but the single-view design may underperform in highly diverse geometric conditions. Second, AWU-OBB mainly covers representative single-type adverse weather conditions, whereas real UAV missions more often involve multiple degradation factors occurring simultaneously or changing continuously during flight. Therefore, the current benchmark still has room for further extension in terms of scene complexity. Third, the dynamic dilation rates in LPM are still determined by predefined rules, and their adaptability under varying degradation levels and target scales could be further improved.

These limitations are also reflected in the qualitative results, where missed detections and imperfect localization may still occur in scenes with dense occlusion, strong reflection interference, or extremely weak local contrast.

Moreover, all experiments in this work were conducted under a unified input resolution of 640×640 for fair comparison and computational consistency. Although this setting is widely adopted in YOLO-based detectors, it may suppress some small-scale or weak-contrast details in high-resolution UAV imagery, particularly in severely degraded weather scenes.

In addition, the analysis of SELPNet-Lite suggests that the proposed design can be adapted to different computational budgets, which may support future deployment-oriented optimization on UAV platforms.

Future work may proceed in three directions. First, more adaptive geometric modeling strategies, such as content-aware sampling mechanisms or attention-guided affine prediction, could be explored to improve the capability of SEC under complex rotation, scale variation, and viewpoint disturbance. Second, AWU-OBB could be extended to more realistic compound degradation scenarios, and physics-based atmospheric models could be incorporated to support more targeted data augmentation and training. Third, further investigation of structured pruning and knowledge distillation techniques applied to the full SELPNet could yield additional lightweight variants that better bridge the accuracy gap between SELPNet-Lite and the full model. These directions may help improve the robustness, adaptability, and deployment flexibility of future UAV object detection systems under adverse weather conditions.

Future work may also investigate standardized restoration-plus-detection cascades under unified latency constraints, so as to enable a more systematic comparison between preprocessing-based enhancement strategies and detector-side robustness design for adverse-weather UAV perception.

## 6. Conclusions

This study addresses UAV oriented object detection under adverse weather conditions and proposes SELPNet, an improved detection framework built upon YOLOv13-n. To handle geometric instability and contextual information loss caused by weather-induced degradation, two complementary modules are introduced. The Scene Exploration Convolution (SEC) enhances geometric representation stability under rotation and scale variation, while the Large Format Perception Module (LPM) improves large-range contextual perception and semantic compensation. In addition, the AWU-OBB dataset is constructed and released as a dedicated benchmark for UAV oriented object detection in adverse weather scenarios.

Experimental results demonstrate that the proposed method achieves better overall performance on the AWU-OBB benchmark than the compared methods and provides a clear improvement over the YOLOv13-n baseline. The ablation study further shows that SEC and LPM play complementary roles: SEC mainly strengthens geometric robustness, whereas LPM mainly improves contextual modeling capability. Their collaboration jointly enhances detection accuracy in complex meteorological environments. Meanwhile, the proposed framework maintains a relatively compact model size and computational cost, indicating its potential for further deployment-oriented optimization on UAV platforms.

Overall, SELPNet and AWU-OBB provide an effective research framework for UAV oriented object detection under adverse weather conditions. The present study verifies the feasibility of improving detection performance from the two perspectives of geometric robustness and contextual perception and also establishes a basis for future work on lightweight implementation, compound degradation modeling, and adaptation to more complex real-world environments.

## Figures and Tables

**Table 1:** Summary of Related Methods.

Category	Core Idea	Models	Advantages	Limitations
**Adverse Weather Perception Methods**				
Restoration	Leverage atmospheric priors or Generative Adversarial Networks (GANs) for pixel-level visual recovery.	IA-YOLO [12], ELS-YOLO [13]	Enhances visual interpretability and masked semantic features in low-visibility.	High inference latency due to cascaded structures; prone to blurring fine edges.
Domain Learning	Utilize semi-supervised transfer or alignment to minimize distribution shifts between domains.	Munir et al. [11], Kalwar et al. [10]	Reduced dependency on real labels; enhances generalization resilience across scenes.	Neglects the coupling between local geometric distortion and aerial viewpoints.
Robust Arch	Design intrinsic noise-resilient operators or disentangle environmental noise in the spectral domain.	TPH-YOLOv5++ [4], Frequency-domain disentanglement [1]	Strong feature preservation under dynamic clutter without preprocessing overhead.	Lack of intrinsic rotation equivariance; sensitive to dynamic perspective variations.
**Feature-Driven Detection Methods**				
Attention-based	Capture long-range pixel dependencies and cross-channel spatial correlations via position encoding.	Gold-YOLO [3], SOAM Block [24], CoordAttn [23]	Excellent discriminative power in cluttered backgrounds; captures multi-scale context.	Prohibitive parameter redundancy; high GFLOPs unsuitable for embedded edge deployment.
Alignment-based	Coordinate feature distributions of sub-tasks via task-aware heads to mitigate dissonance.	TOOD [14], YOLOX [15]	Improved localization precision; alleviates task conflicts in one-stage detectors.	Insufficient robustness against arbitrary rotation; severe loss during feature downsampling.
Efficiency-driven	Optimize information flow and feature pyramids using programmable gradient information (PGI).	YOLOv10 [27], YOLOv9 [25], YOLOv6 [26]	SOTA real-time inference speed; high hardware utilization for on-board processing.	Presence of “information bottleneck”; low recall for densely distributed small targets.

**Table 2:** Detailed scene-wise statistics of AWU-OBB.

Scene Category	Images	Annotated Vehicles	Train	Val	Test
Clear (Standard Urban)	7278	95,342	5081	779	1418
Fog-Haze (Low-Visibility)	4550	58,695	3182	488	880
Nighttime Rain (Specular)	4546	58,189	3176	491	879
Snowfall (Visual Noise)	1821	24,166	1241	189	391
Total	18,195	236,392	12,680	1947	3568

**Table 3:** Detailed hyperparameter settings and experimental configurations.

Category	Hyperparameter/Hardware	Value
Hardware	GPU/VRAM	NVIDIA RTX 4090D/24 GB
	CPU/RAM	AMD EPYC 9754/60 GB
Software	OS/Programming Language	Ubuntu 22.04/Python 3.12
	Deep Learning Framework	PyTorch 2.5.1/CUDA 12.4
Training	Input Resolution	640×640
	Epochs/Batch Size	200/16
	Optimizer	SGD
	Initial Learning Rate ($lr_{0}$)	0.01
	Weight Decay/Momentum	0.0005/0.937
Detection	R-NMS IoU Threshold	0.7
	Confidence Threshold	0.25

**Table 4:** Performance comparison of object detection methods on the AWU-OBB dataset. **Bold** denotes the best detection accuracy (mAP50, mAP, mAP75) in each column.

Method	Backbone	mAP50 (%)	mAP (%)	mAP75 (%)	FPS	Params (M)	FLOPs (G)
**One-stage detectors**							
RetinaNet [31]	ResNet-50	52.14	36.83	47.29	44.6	36.3	88.5
RetinaNet [31]	ResNet-101	54.37	38.52	49.18	38.2	55.2	128.7
TOOD [14]	ResNet-50	56.73	41.02	52.18	38.2	32.0	79.6
TOOD [14]	ResNet-101	58.91	42.78	54.36	32.6	51.0	119.3
FCOS [32]	ResNet-50	53.82	38.14	48.67	47.3	32.1	64.7
FCOS [32]	ResNet-101	55.48	39.67	50.33	41.5	48.7	95.4
IA-YOLO [12]	CSPDarknet53	60.22	43.19	55.81	52.3	46.2	72.1
TPH-YOLOv5 [2]	CSPDarknet53	62.91	45.38	57.34	35.7	86.5	108.3
ELS-YOLO [13]	EfficientNet-B3	60.87	43.96	55.97	49.1	31.6	58.7
Gold-YOLO [3]	CSPDarknet53	63.47	46.12	58.73	38.4	72.8	96.2
TPH-YOLOv5++ [4]	CSPDarknet53	64.05	46.78	59.42	33.8	92.3	115.6
**Two-stage and query-based detectors**							
Faster R-CNN [33]	ResNet-50	48.23	33.06	43.41	29.8	41.4	91.2
Faster R-CNN [33]	ResNet-101	50.36	34.71	45.12	25.4	60.1	134.8
Cascade R-CNN [34]	ResNet-50	55.71	40.12	51.46	26.3	69.2	107.5
Cascade R-CNN [34]	ResNet-101	57.84	41.93	53.27	22.7	88.6	150.3
DINO [35]	ResNet-50	62.13	44.87	57.62	26.5	47.3	116.9
DINO [35]	ResNet-101	63.87	46.23	59.14	22.1	66.5	157.3
**YOLO-based detectors**							
YOLOv6 [26]	EfficientRep	59.43	42.56	54.81	68.3	17.2	44.9
YOLOv8-n [36]	CSPDarknet	64.38	47.03	59.14	62.7	3.27	8.7
YOLOv9-n [25]	CBNet	65.12	47.81	60.03	58.4	2.70	7.9
YOLOv10-n [27]	CSPDarknet	65.84	48.26	60.71	63.1	2.73	6.7
YOLOv11-n [37]	CSPDarknet	66.53	48.92	61.18	61.5	2.84	7.2
YOLOv26-n [38]	CSPDarknet	70.18	51.37	61.02	57.8	2.81	7.1
Baseline: YOLOv13-n [39]	CSPDarknet	69.04	50.14	59.32	55.42	2.45	6.4
**SELPNet (Ours)**	CSPDarknet	**73.30**	**54.22**	**63.62**	32.69	2.56	6.6

**Table 5:** Ablation study of SEC and Dynamic Dilation on YOLOv13-Nano.

Setting	Description	mAP50	ΔmAP50	mAP	mAP75	Params (M)	FLOPs (G)	FPS
A	YOLOv13-Nano	69.04	–	50.14	59.32	2.45	6.4	55.42
B	+SEC	71.79	+2.75	52.57	62.53	2.55	6.6	34.83
C	+LPM	72.74	+3.70	53.87	63.34	2.46	6.4	55.65
D	SEC + LPM (Ours)	73.30	+4.26	54.22	63.62	2.56	6.6	32.69

**Table 6:** Ablation study on rotation quantity ($N_{rot}$) and maximum rotation angle ($\theta_{max}$). The default settings are marked in **bold**.

Variant	Configuration	mAP50 (%)	ΔmAP50
$N_{rot}=1$	Single rotation view	57.10	−4.90
**$N_{\mathrm{rot}}=2$ (default) **	**Dual rotations (Ours)**	**62.00**	**–**
$N_{rot}=4$	Quadruple rotations	56.80	−5.20
$N_{rot}=8$	Octuple rotations	58.40	−3.60
$\theta_{max}=15{}^{\circ}$	Narrow range	55.30	−6.70
**$\theta_{\max}=30{}^{\circ}$ (default)**	**Moderate range (Ours)**	**62.00**	**–**
$\theta_{max}=45{}^{\circ}$	Wide range	56.60	−5.40
$\theta_{max}=90{}^{\circ}$	Orthogonal range	52.90	−9.10

**Table 7:** Ablation study on kernel size (*k*) and dilation strategy. The default settings are marked in **bold**.

Variant	Configuration	mAP50 (%)	ΔmAP50
***A: Kernel Size***			
k=3	Small receptive field	61.80	−0.64
k=5	Medium receptive field	61.90	−0.54
**k=7 (default)**	**Large receptive field (Ours)**	**62.44**	**–**
k=9	Extra large receptive field	62.70	+0.26
***B: Dilation Strategy***			
Static	Fixed dilation (d=1)	59.20	−3.24
**Linear (default)**	**Linearly increasing (1,2,3…) (Ours)**	**62.44**	**–**
Sparse	Sparsely increasing (1,3,5…)	60.30	−2.14
Exponential	Exponentially increasing (1,2,4…)	60.90	−1.54

**Table 8:** Scene-wise detection performance on the AWU-OBB test set.

Scene Category	Test	YOLOv13-n			SELPNet (Ours)		
		mAP	mAP50	mAP75	mAP	mAP50	mAP75
Clear (Standard Urban)	1418	73.10	53.70	63.20	76.50	58.00	67.60
Fog-Haze (Low-Visibility)	880	67.90	49.10	58.00	72.80	53.30	62.40
Nighttime Rain (Specular)	879	67.20	48.80	57.70	72.20	52.70	62.40
Snowfall (Visual Noise)	391	60.70	42.60	51.90	65.30	46.10	55.20
Weighted average	3568	69.04	50.14	59.32	73.30	54.22	63.62

**Table 9:** Comparison of SELPNet-Lite and the full SELPNet with the YOLOv13-n baseline. The best results are marked in **bold**.

Method	mAP50 (%)	mAP (%)	mAP75 (%)	Params (M)	FLOPs (G)	FPS
YOLOv13-n (Baseline)	69.04	50.14	59.32	2.45	6.4	55.42
SELPNet-Lite	71.51	52.14	61.32	2.38	6.2	58.73
SELPNet (Full)	**73.30**	**54.22**	**63.62**	2.56	6.6	32.69

**Table 10:** Inference time and accuracy comparison between SELPNet and representative detection frameworks on the AWU-OBB dataset. Time Ratio denotes the relative inference time compared to SELPNet. Our method achieves the highest mAP50 while maintaining acceptable latency.

Framework	Backbone	mAP (%)	mAP50 (%)	Time (ms)	Time Ratio
**One-stage detectors**					
RetinaNet [31]	ResNet-50	36.83	52.14	22.42	0.73×
RetinaNet [31]	ResNet-101	38.52	54.37	26.18	0.86×
FCOS [32]	ResNet-50	38.14	53.82	21.14	0.69×
FCOS [32]	ResNet-101	39.67	55.48	24.10	0.79×
TOOD [14]	ResNet-50	41.02	56.73	26.18	0.86×
TOOD [14]	ResNet-101	42.78	58.91	30.67	1.00×
IA-YOLO [12]	CSPDarknet53	43.19	60.22	19.12	0.63×
TPH-YOLOv5 [2]	CSPDarknet53	45.38	62.91	28.01	0.92×
ELS-YOLO [13]	EfficientNet-B3	43.96	60.87	20.37	0.67×
Gold-YOLO [3]	CSPDarknet53	46.12	63.47	26.04	0.85×
TPH-YOLOv5++ [4]	CSPDarknet53	46.78	64.05	29.59	0.97×
**Two-stage and query-based detectors**					
Faster R-CNN [33]	ResNet-50	33.06	48.23	33.56	1.10×
Faster R-CNN [33]	ResNet-101	34.71	50.36	39.37	1.29×
Cascade R-CNN [34]	ResNet-50	40.12	55.71	38.02	1.24×
Cascade R-CNN [34]	ResNet-101	41.93	57.84	44.05	1.44×
DINO [35]	ResNet-50	44.87	62.13	37.74	1.23×
DINO [35]	ResNet-101	46.23	63.87	45.25	1.48×
**YOLO-based lightweight detectors**					
YOLOv6 [26]	EfficientRep	42.56	59.43	14.64	0.48×
YOLOv8-n [36]	CSPDarknet	47.03	64.38	15.95	0.52×
YOLOv9-n [25]	CBNet	47.81	65.12	17.12	0.56×
YOLOv10-n [27]	CSPDarknet	48.26	65.84	15.85	0.52×
YOLOv11-n [37]	CSPDarknet	48.92	66.53	16.26	0.53×
YOLOv26-n [38]	CSPDarknet	51.37	70.18	17.30	0.57×
YOLOv13-n [39]	CSPDarknet	50.14	69.04	18.05	0.59×
SELPNet-Lite (Ours)	CSPDarknet	52.14	71.51	17.03	0.56×
**SELPNet (Ours)**	CSPDarknet	**54.22**	**73.30**	**30.59**	**1.00×**
**Large Backbone-based detectors**					
Cascade R-CNN [34]	Swin-L	53.41	72.08	152.95	5.00×
DINO [35]	Swin-L	53.67	72.35	122.36	4.00×

**Table 11:** Edge-device deployment results on Raspberry Pi 4. Representative methods from each category are selected for comparison. All models are exported to ONNX format and executed via ONNX Runtime (CPU). Input resolution: 640×640. Time Ratio denotes the relative inference time compared to YOLOv13-n on the Raspberry Pi 4.

Framework	Backbone	mAP50 (%)	Params (M)	RPi4 Time (s)	Time Ratio
**One-stage detectors**					
TOOD [14]	ResNet-101	58.91	51.0	12.05	8.73×
TPH-YOLOv5++ [4]	CSPDarknet53	64.05	92.3	18.91	13.70×
**Two-stage detectors**					
Cascade R-CNN [34]	ResNet-101	57.84	88.6	22.73	16.47×
DINO [35]	ResNet-101	63.87	66.5	19.48	14.12×
**YOLO-based lightweight detectors**					
YOLOv13-n [39]	CSPDarknet	69.04	2.45	1.38	**1.00×**
SELPNet-Lite (Ours)	CSPDarknet	71.51	2.38	1.31	0.95×
**SELPNet (Ours)**	CSPDarknet	**73.30**	2.56	**1.52**	1.10×
**Large Backbone-based detectors**					
Cascade R-CNN [34]	Swin-L	72.08	253.7	68.42	49.58×
DINO [35]	Swin-L	72.35	218.4	54.19	39.27×

## Data Availability

The AWU-OBB dataset generated and analyzed during the current study is available from the corresponding author upon reasonable request. The source code for SELPNet will be publicly released upon acceptance of the manuscript. The other datasets used for comparative experiments, including DOTA, HRSC2016, and DIOR-R, are publicly available and have been properly cited in the text.

## Abbreviations

The following abbreviations are used in this manuscript:

UAV	Unmanned Aerial Vehicle
OBB	Oriented Bounding Box
SEC	Scene Exploration Convolution
LPM	Large Format Perception Module
DSConv	Depthwise Separable Convolution
C2f	Cross Stage Partial with two-fold split
BN	Batch Normalization
R-NMS	Rotated Non-Maximum Suppression
DFL	Distribution Focal Loss
mAP50	mean Average Precision
FLOPs	Floating Point Operations
GFLOPs	Giga Floating Point Operations
SGD	Stochastic Gradient Descent
RF	Receptive Field
mAP	mean Average Precision
mAP50	mean Average Precision at IoU threshold of 0.50
mAP75	mean Average Precision at IoU threshold of 0.75
